# Alkali-Activated Binders as Sustainable Alternatives to Portland Cement and Their Resistance to Saline Water

**DOI:** 10.3390/ma17174408

**Published:** 2024-09-06

**Authors:** Erion Luga, Enea Mustafaraj, Marco Corradi, Cengiz Duran Atiș

**Affiliations:** 1 College of Engineering and Technology, American University of the Middle East, Egaila 54200, Kuwait; erion.luga@aum.edu.kw (E.L.); enea.mustafaraj@aum.edu.kw (E.M.); 2 Department of Engineering and Technology, University of Huddersfield, Huddersfield HD1 3DH, UK; 3 Department of Civil Engineering, Erciyes University, Kayseri 38039, Turkey; cdatis@erciyes.edu.tr

**Keywords:** alkali-activated binders, geopolymer, wet–dry cycles, durability

## Abstract

Alkali-activated binders have emerged as promising alternatives to Ordinary Portland Cement (OPC) due to their sustainability features and potential advantages. This study evaluates the durability properties of heat-cured fly ash (FA) and ground granulated blast-furnace slag (GGBFS) geopolymer mortars activated with sodium hydroxide, which were subjected to wet–dry cycling in saline environments. Three series of FA, a FA/GGBFS blend, and GGBFS mortars previously optimized on a compressive strength basis were investigated and compared against two control OPC mixes. Performance indicators such as the water absorption, porosity, flexural strength, and compressive strength were analyzed. The results demonstrate that geopolymer mortars have significantly reduced water absorption and porosity with increasing wet–dry cycles. The compressive strength of the FA/GGBFS mortars also increased from 66.5 MPa (untreated) to 87.9 MPa over 45 cycles. The flexural strength remained stable or improved slightly across all geopolymer mortars. The control OPC specimens experienced significant deterioration, with compressive strength in CEM I 42.5R dropping from 51.8 to 17.1 MPa. These findings highlight the superior durability of geopolymer mortars under harsh saline conditions, demonstrating their potential as a resilient alternative for coastal and marine structures.

## 1. Introduction

**Figure 1:**
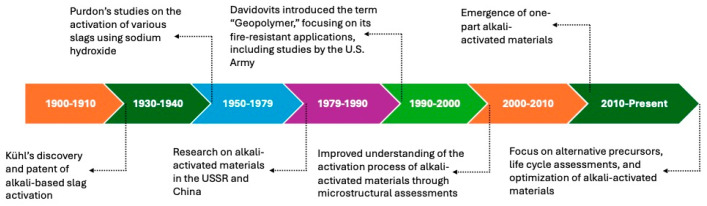
Historical development of AAMs [4].

The cement industry is a significant contributor to global carbon dioxide emissions, accounting for approximately 7% of the world’s CO_2_ footprint, and is a substantial consumer of natural resources and energy [1]. This challenging backdrop has spurred global research into alternative binding materials capable of substituting Ordinary Portland Cement (OPC), among which alkali-activated binders (AABs) have surfaced as an interesting alternative. According to Ouellet-Plamondon and Habert [2], only some of these materials offer significant reductions in CO_2_ emissions and energy consumption in production, and Provis et al. [3] emphasized that geopolymers “are not intrinsically or fundamentally ‘low-CO_2_’ unless designed effectively to achieve such performance”. Their development can be traced back to early studies on slag cement in the early 20th century (Figure 1). Alkali-activated materials (AAMs) can be categorized based on their chemical composition into high-calcium, low-calcium, and blended systems, each requiring different curing regimes to optimize their performance. High-calcium systems, which include precursors like blast-furnace Slag (BFS) and class C fly ash, typically achieve higher initial strength and lower setting times but are prone to shrinkage and porosity if not properly cured. In contrast, low-calcium systems, such as those based on metakaolin, require higher temperatures for curing to attain adequate strength [4]. However, blending fly ash and slag eliminates the need for elevated curing temperatures as well as delays fast setting [5,6].

Alkali activation, a transformative process that involves the reaction of amorphous alumina and silica with strong alkaline solutions such as sodium hydroxide (NaOH), potassium hydroxide (KOH), and water glass, has shown significant potential in the construction industry. This method capitalizes on industrial byproducts, including slag and fly ash, turning these potential environmental liabilities into valuable precursors for construction materials [7,8]. By utilizing these byproducts, alkali activation not only helps in waste management but also contributes to the production of sustainable and high-performance construction materials. AABs excel where traditional concretes fail, offering superior resistance to harsh conditions, including high temperatures, acidic environments, sulfate attack, and seawater exposure, thus demonstrating their enhanced durability [9,10,11,12,13,14,15,16,17]. Li et al. [18] evidenced the robustness of alkali-activated slag mortars under aggressive conditions, resulting in a minimal impact of wet–dry cycling on compressive and flexural strengths [17]. This discovery [18], coupled with Monticelli et al.’s [19] finding that alkali-activated fly ash mortars offer improved corrosion protection for steel reinforcements, marks a significant advancement in the pursuit of more resilient construction materials. Further investigations highlighted the adaptability of alkali-activated materials to marine conditions, showcasing their early strength development and low absorption despite exposure to acidic environments.

The exploration of durability under cyclic wet–dry conditions extends to understanding the complex behaviors of conventional concrete and mortar [20]. Studies such as those by Sun et al. [21] and Zhang et al. [22] shed light on the nuanced changes in concrete’s compressive strength and water absorption under such cycles, suggesting a toughening effect that eventually gives way to material degradation. Yang et al. [23] investigated the feasibility of employing AABs by utilizing a blend of artificial seawater, sea sand, Coral Coarse Aggregate (CCA), Normal Limestone Aggregate (NLA), and slag-based AAMs, which included 15 wt% fly ash and 5 wt% silica fume to develop performance-based alkali-activated seawater sea-sand concrete (ASSC). The use of CCA contributed to a denser interfacial transition zone (ITZ) with fewer microcracks, as well as improved mechanical performance and durability [22].

On the other hand, Dehestani et al. [24] observed a reduction in fracture toughness resulting from wet–dry cycling, a finding echoed by Noor-E-Khuda [25], who reported declining compressive and flexural strengths and bond strength between cement mortar and CFRP with repeated cycles. These studies provide critical insights into the deterioration mechanisms that affect materials under cyclic environmental stress, establishing a foundation for future research to enhance material resilience.

While extensive research has been conducted on the performance of alkali-activated binders, the specific response of these materials to cyclic wet–dry conditions in saline environments remains less explored. This gap is particularly significant given the potential applications of these materials in coastal and marine construction, where exposure to harsh saline conditions is a critical factor. The current study seeks to address this gap by examining the durability characteristics of geopolymer mortars—comprised of heat-cured fly ash (FA) and ground granulated blast-furnace slag (GGBFS)—under such conditions. By comparing these to conventional OPC mortars, this research will provide valuable insights into the potential of alkali-activated binders to serve as robust, sustainable alternatives in coastal and marine construction.

## 2. Experimental Program

### 2.1. Materials

This study aimed to evaluate the resistance of heat-cured geopolymer mortars made from FA and slag and activated with sodium hydroxide when exposed to saline water. Initially, the study modeled and tested three optimal alkali-activated fly ash/slag mortar mixes [17]. These geopolymer mortars were then subjected to cyclic wet–dry cycles in salty water and compared with two different control mixes produced from OPC types CEM I 42.5R and CEM I 52.5R. The analysis included assessments of the water absorption, porosity, and both flexural and compressive strengths under wet–dry cycling. The performance of the geopolymer mortars was compared to that of the control mixes to determine their durability.

The GGBFS used in this study was obtained from the Iskenderun Demir ve Celik steel Factory (Iskenderun, Turkey), whereas the FA was from the Sugozu thermal power plant (Adana, Turkey). The materials have a specific gravity of 2.81 g/cm^3^ and 2.39 g/cm^3^, respectively. Blaine’s specific surface is 4250 cm^2^/g and 2900 cm^2^/g. According to ASTM C989 [26], the slag is classified as a grade of 80 slag. The FA has a content of SiO_2_ + Al_2_O_3_ + Fe_2_O_3_ > 70%, CaO < 10% according to EN 450-1 [27], and 28-day pozzolanic activity of 78 according to ASTM C618-94a [28]. Table 1 shows the chemical compositions of GGBFS and FA.

The CEM I 42.5 R and CEM I 52.5 R were produced in accordance with EN 197-1 [29]. CEM I 42.5R was produced in the Baştaş cement factory (Elamadag, Ankara, Turkey), whereas the CEM I 52.5 R Super white cement was produced in the Çimsa (Turkey) cement factory in Mersin, Turkey.

### 2.2. Preparation of Mortar Samples and Test Methods

Mortar samples were prepared and cast into prismatic molds measuring 40 × 40 × 160 mm in accordance with the EN 196-1 standard [30]. Once they were placed in three-gang steel molds, the geopolymer mortars were heat-cured in an oven at 100 °C for 72 h. This curing method was chosen for its economic feasibility and appropriateness for NaOH-based systems [9], whereas the curing temperature was defined based on the study of Luga and Atis, suggesting 100 °C as the correct temperature to achieve the highest performance for the given case [9]. After curing, the mortars were allowed to cool to room temperature before being removed from the molds for testing.

In contrast, the OPC control mortars were cured by being immersed in water for 28 days, in line with EN 196-1 [30]. The materials for the mortar specimens were weighed separately according to the prescribed ratios on a precision scale. Table 2 outlines the mix design and curing conditions for the mortars.

The flexural strength of the mortar samples was measured according to the EN 1015-11 standard [31]. The tests were conducted on 40 × 40 × 160 mm prismatic specimens under three-point bending loading, with a 100 mm distance between end supports. The average flexural tensile strength was calculated based on three specimens.

The compressive strength of the mortar samples was assessed in compliance with EN 196-1 [30]. The mortar specimens were tested using a loading rate of 500 N/s and a loading area of 40 × 40 mm. The average value from six test specimens was taken as the compressive strength.

The physical properties of the samples were determined by measuring the Oven-Dry weight (OD), the water-Saturated Surface-Dry weight (SSD), and the weight when Immersed in Water (IW) using the Archimedes scale. These measurements were used to calculate the unit weight, water absorption, and porosity of the mortars. Water absorption and porosity were calculated using the weight values in different conditions, such as (SSD-OD)/OD and (SSD-OD)/(SSD-IW), respectively.

The wet–dry treatment was designed to simulate tidal events and assess the geopolymer mortars’ wetting and drying resistances. Before starting the cycles, the dry, water-saturated, and immersed in water weights of the samples were measured. A 5% concentration by weight of saline solution (NaCl) was prepared, and the samples were subjected to 12 h of immersion at room temperature, followed by 12 h of drying at 105 °C for a more aggressive testing environment. The samples underwent 15, 30, and 45 wet–dry cycles across different series. The physical and mechanical properties of the mortars were measured on a total of 20 series corresponding to 5 mortar types (FA/GGBFS, FA, GGBFS, CEM I 42.5R, and CEM I 52.5R) and four durations of the aging treatment (0, 15, 30, and 45 cycles). The total number of samples tested was 60 for water absorption tests, 60 for porosity tests, 60 for flexural testing, and 120 for compressive testing, for a total of 300 samples tested.

## 3. Results and Discussion

The test results offer insights into the performances and durabilities of different types of mortars when exposed to varying wet–dry cycles in a saline environment. The analysis and the results are limited to the specific types of mortars tested, and the reader should be aware of this limitation. However, the large number of tests conducted and the different types of mortars considered can provide some interesting insights into the mechanical behaviors of certain sustainable mortars when exposed to saline water environments.

The data include the water absorption, porosity, flexural strength, and compressive strength for three types of alkali-activated mortars (FA/GGBFS, FA, and GGBFS) and two types of traditional cement mortars (made with CEM I 42.5R and CEM I 52.5R OPC). By comparing the performances of these mortars across 0, 15, 30, and 45 wet–dry cycles, the study evaluates how the prolonged exposure to wet–dry cycles affects the durability and mechanical properties of each type of mortar in a saline solution. The findings reveal distinct trends in the performances of the various materials, providing valuable insights into the potential advantages and limitations of each mortar type under challenging environmental conditions (Table 3).

**Figure 2:**
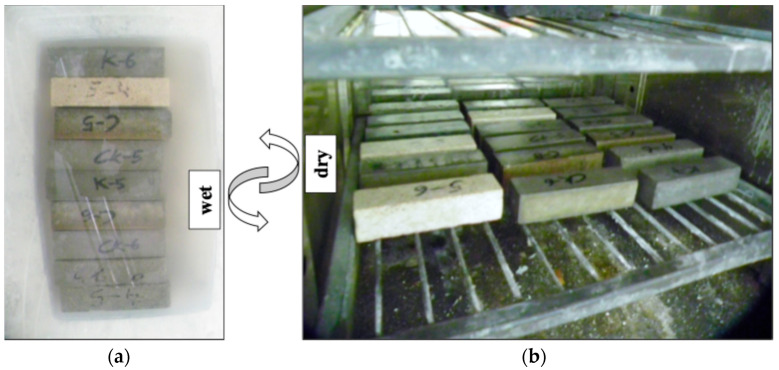
(**a**) Samples under saline treatment. (**b**) Drying of mortar specimen for wet–dry treatment.

The water absorption results provide insights into how different mortars react to wet–dry cycles in a saline environment. Water absorption is a critical parameter for understanding mortar durability and longevity, particularly in environments with frequent water and humidity variations. The results of all five mortar types are summarized in Figure 2.

The initial water absorption rate for the FA/GGBFS mortars was 6.8% at zero cycles. After undergoing 15 wet–dry cycles, the water absorption increased to 7.6%, suggesting an initial degradation in resistance to water. However, as the number of wet–dry cycles increased, the water absorption decreased significantly. By 30 cycles, the water absorption rate fell to 5.8% and further decreased to 4.7% after 45 cycles. This trend indicates that the FA/GGBFS mortars become more resistant to water absorption with prolonged exposure to wet–dry cycles in salty water.

In the case of geopolymer mortars, the water absorption values initially exhibit an increase due to leaching. Later, the Na+ starts to react with the excess silica and alumina. In the case of OPC mortars, the lack of excess silica and alumina polymerization does not occur, but the NaCl precipitates in the pores, thus decreasing the water absorption.

The FA mortars initially absorbed 5.7% of water at zero cycles. Following 15 cycles, the water absorption increased to 6.6%, similar to the trend observed in the FA/GGBFS mortars. However, as the number of cycles progressed, the water absorption decreased substantially. At 30 cycles, the water absorption rate dropped to 4.0%, further declining to 3.8% after 45 cycles. These results suggest that FA mortars improve their water absorption resistance with more cycles.

The GGBFS mortars showed an initial water absorption rate of 4.9% at zero cycles. After 15 cycles, the water absorption increased to 6.2%, indicating a decline in their performance. Nonetheless, like the other types of mortars, the GGBFS mortars demonstrated an improvement over time. At 30 cycles, the water absorption rate dropped to 3.9%, and it decreased further to 2.6% after 45 cycles. This trend highlights the enhanced resistance to water absorption as GGBFS mortars undergo more cycles.

The control OPC mortars, made with CEM I 42.5R and CEM I 52.5R cements, exhibited similar trends in terms of water absorption. The CEM I 42.5R mortars had an initial water absorption rate of 7.9% at 0 cycles, which decreased to 3.6% after 15 cycles and stabilized at around 3.9% for the subsequent cycles. Increasing the strength class of the OPC mortar did not change this trend: the CEM I 52.5R mortars initially absorbed 7.6% of water at 0 cycles, and this decreased to 4.1% after 15 cycles, further declining to 3.4% after 30 cycles and 2.9% after 45 cycles. However, the test results demonstrate that the OPC mortars exhibited the highest reduction in water absorption, with a reduction percentage of 62–65% at the end of the treatment.

All three types of alkali-activated mortars (FA/GGBFS, FA, and GGBFS) experienced maximum water absorption values at 15 cycles (approximately ranging between 13 and 16%). This peak is followed by a consistent decrease in water absorption over the remaining cycles. This pattern indicates that while water absorption initially increases, possibly due to early exposure effects, the mortars adapt and become more resistant to moisture and water over time.

This trend of declining water absorption after 15 cycles suggests that the alkali-activated mortars improve their durability and resistance to moisture exposure through prolonged wet–dry cycles in a saline environment.

**Figure 3:**
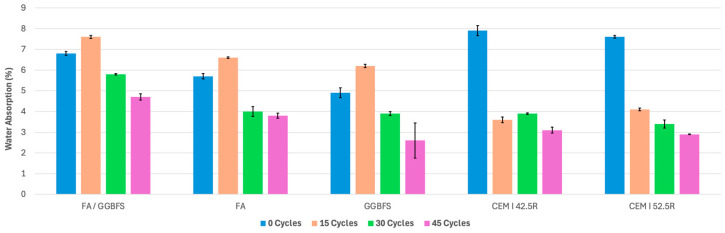
Water absorption results under wet–dry cycling.

**Figure 4:**
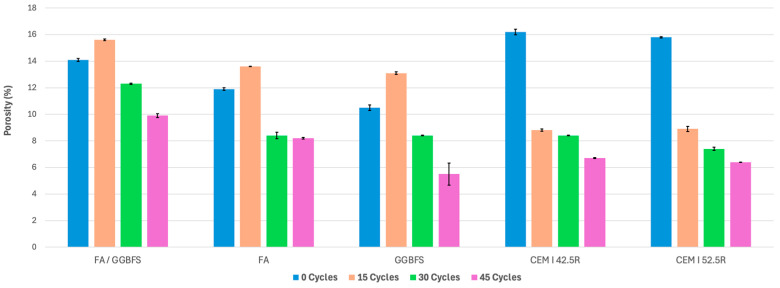
Porosity values under wet–dry cycling.

The porosity results also offer an initial understanding of different mortars’ internal structures and potential durability when subjected to wet–dry cycles in a saline environment. Porosity is a crucial factor influencing the strength and resistance of mortars to environmental actions. The porosity test results are summarized in Figure 3. For the FA/GGBFS mortars, the initial porosity at zero cycles was 14.1%. After 15 wet–dry cycles, the porosity increased to 15.6%, suggesting a decline in resistance to environmental actions. As the number of cycles increased, the porosity showed a significant decline. By 30 cycles, the porosity dropped to 12.3% and further decreased to 9.9% after 45 wet–dry cycles, indicating improved structural integrity and a gradual impermeability of the mortar (Figure 4).

The FA mortars initially exhibited a porosity of 11.9% at zero cycles. Following 15 cycles, the porosity increased to 13.6%, similar to the FA/GGBFS mortars. Nonetheless, the porosity gradually decreased with more cycles, reaching 8.4% at 30 cycles and 8.2% after 45 cycles. This reduction demonstrates that FA mortars improve their internal structure and undergo a decrease in porosity as the number of cycles increases.

The GGBFS mortars exhibited an initial porosity of 10.5% at zero cycles. After undergoing 15 cycles, the porosity increased to 13.1%, indicating a temporary performance decrease. Despite this, the mortars showed consistent improvement over time, with the porosity falling to 8.4% at 30 cycles and further dropping to 5.5% after 45 cycles. These results suggest that GGBFS mortars become less porous and more durable with aging wet–dry cycles.

The control OPC mixes CEM I 42.5R and CEM I 52.5R demonstrated varying trends in porosity. The CEM I 42.5R mortars had an initial porosity of 16.2% at 0 cycles, which significantly decreased to 8.8% after 15 cycles and remained stable around 8.4% for the subsequent cycles. The CEM I 52.5R mortars began with a porosity of 15.8% at 0 cycles, which decreased steadily over time, falling to 8.9% after 15 cycles, 7.4% after 30 cycles, and 6.4% after 45 cycles.

All three types of alkali-activated mortars (FA/GGBFS, FA, and GGBFS) experienced maximum porosity values at 15 cycles. This is followed by a consistent decrease in the porosity over the remaining cycles. This behavior suggests that while the porosity initially increases due to early exposure effects, the mortars adapt and become less porous over time due to the crystallization of Na-based salts inside the pores of the mortars. The declining porosity after 15 cycles indicates that the alkali-activated mortars improve their internal structure and resistance to moisture and environmental stressors with prolonged exposure to wet–dry cycles in a saline environment.

Changes in the porosity are likely influenced by microstructural changes, chemical reactions, and the material properties of the mortars. Alkali-activated mortars benefit from the formation of dense internal structures and reduced moisture absorption, leading to decreased porosity over time. While also showing decreased porosity, traditional cement mortars may face challenges due to environmental degradation. These results emphasize that as mortars undergo more aging cycles, they tend to develop stronger internal structures, reducing their porosity and enhancing their durability, which is essential for enduring harsh environmental conditions.

**Figure 5:**
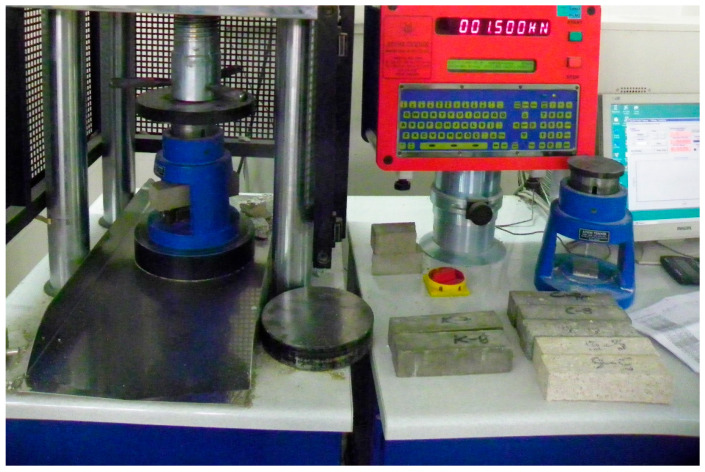
Flexural test of mortar prisms.

**Figure 6:**
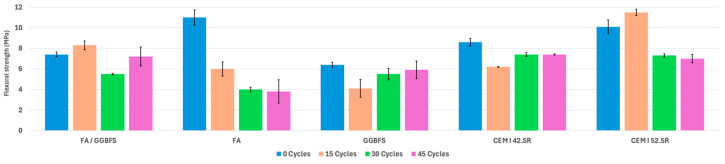
Flexural strength values under wet–dry cycling.

Regarding flexural strength, a key parameter that assesses a mortar’s ability to endure shear and tensile stresses, several interesting observations can be drawn from the test results. The mortar prisms were tested, as shown in Figure 5, and the summary of the results is illustrated in Figure 6.

For FA/GGBFS mortars, wet–dry cycling in a saline environment did not cause significant strength reductions: the initial flexural strength was 7.4 MPa at 0 cycles, and after undergoing 15 wet–dry cycles, it increased to 8.3 MPa. However, after 30 cycles, it decreased to 5.5 MPa, and by 45 cycles, it recovered to 7.2 MPa.

On the contrary, wet–dry cycling in a saline environment produced a significant reduction in the bending strength of FA mortars, up to 65%; these mortar specimens exhibited an initial flexural strength of 11.0 MPa at zero cycles. After 15 cycles, the flexural strength decreased significantly to 6.0 MPa, revealing a reduction in the performance. As the number of cycles progressed, the flexural strength further declined to 4.0 MPa at 30 cycles and 3.8 MPa at 45 cycles, suggesting a consistent decline in the mortar’s structural bending capability under cyclic stress. Clearly, this is an indication of severe mechanical degradation produced by the saline treatment.

The GGBFS mortars had an initial, in untreated conditions, flexural strength of 6.4 MPa. After 15 cycles, the strength decreased to 4.1 MPa, reflecting a decrease in the performance. Nonetheless, the strength increased to 5.5 MPa at 30 cycles and further to 5.9 MPa after 45 cycles. The variations in the compressive strengths of FA/GGBFS and GGBFS mortars are minor and fall within the expected range of variability for this type of test and material. It is challenging to determine whether the treatment has led to a decrease or an improvement in the compressive strength. The test results should be interpreted with caution due to the limited number of specimens tested and the natural variability in the mechanical properties of the mortars.

The control OPC mixes of CEM I 42.5R and CEM I 52.5R demonstrated different trends in flexural strength. The CEM I 42.5R mortars showed an initial flexural strength of 8.6 MPa at 0 cycles, which decreased to 6.2 MPa after 15 cycles and stabilized at 7.4 MPa for the subsequent cycles. The compressive strength of this lower-grade OPC remained largely unchanged after the saline treatment. Conversely, using a higher strength class of OPC (CEM I 52.5R) in mortars appears to result in a slight reduction in the compressive strength, ranging from 20% to 30%. It is worth noting again that due to the inherent variability in strength measurements from this type of test, the results should be interpreted cautiously. However, the overall trend suggests a clear indication of mechanical degradation following saline cycling treatment. Initially, CEM I 52.5R exhibited a flexural strength of 10.1 MPa at 0 cycles, increasing to 11.5 MPa after 15 cycles but decreasing to 7.3 MPa at 30 cycles and 7.0 MPa after 45 cycles.

Finally, it is necessary to mention a result that applies to all types of mortars tested. The coefficient of variation (COV) of the bending tests was found to be significantly low, with an average value of 0.14. This low COV value increases the reliability of the results obtained, reducing the impact of the natural variability of the experimental outcomes.

The changes in the flexural strengths of different mortars subjected to wet–dry cycling in a saline environment can be attributed to several key factors:

- Chemical interactions and material composition: Chemical reactions in alkali-activated mortars (FA/GGBFS, FA, and GGBFS) lead to the formation of dense polymeric structures, which enhance the flexural strength. The material composition plays a crucial role, as the combination of FA and GGBFS creates a stronger, durable matrix.
- Microstructural changes: Exposure to wet–dry cycles can induce microstructural changes in the mortars. Alkali-activated mortars benefit from these changes, improving their flexural strength over time.
- Moisture absorption and porosity: The ability of mortars to absorb moisture and their porosity influence flexural strength. Alkali-activated mortars, which exhibit lower water absorption and porosity with increased cycles, maintain or improve their flexural strength over time.
- Environmental stressors and degradation: Traditional OPC mortars (CEM I 42.5R and CEM I 52.5R) experience more significant challenges from environmental stressors such as moisture and saline exposure. This can lead to microcracks and deterioration, resulting in a decline in the flexural strength over time. For instance, FA mortars declined significantly in their flexural strength from 6.0 MPa at 15 cycles to 3.8 MPa after 45 cycles.

In summary, the results show variations in the flexural strengths of different mortars as they experience wet–dry cycles. The changes in flexural strength are influenced by the chemical interactions, microstructural changes, and material compositions of different mortars. Alkali-activated mortars tend to maintain or improve their flexural strength due to their dense structure and lower moisture absorption, while traditional OPC mortars face challenges from environmental degradation.

The compressive strength test results provide insights into the abilities of different mortars to withstand compressive loads across varying numbers of wet–dry cycles in a saline environment. Compressive strength is crucial for understanding mortars’ load-bearing capacity and overall durability under challenging conditions.

**Figure 7:**
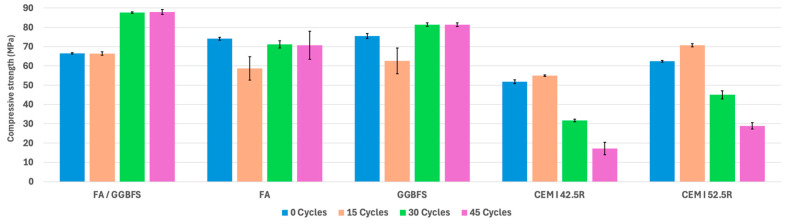
Compressive strength values under wet–dry cycling.

Mortars are commonly used in masonry construction to build walls, whose primary structural function is to withstand compressive stresses from both dead and variable loads. Since masonry is a combination of blocks and mortar, the overall structural properties of the masonry depend on the strength of its constituent materials. Figure 7 summarizes the compressive strength of each specimen.

The results from the compression tests are particularly noteworthy for the sustainable mortars examined in this study. While traditional OPC mortars showed a significant reduction in their compressive strength after saline treatment, indicating serious mechanical degradation, the various types of sustainable mortars demonstrated better resistance to the saline treatment, with less severe mechanical degradation compared to the OPC mortars.

The FA/GGBFS mortars’ initial compressive strength was 66.5 MPa at zero cycles. After undergoing 15 wet–dry cycles, the compressive strength slightly decreased to 66.41 MPa. However, as the number of cycles increased, the compressive strength significantly improved, reaching 87.6 MPa at 30 cycles and remaining relatively high at 87.97 MPa after 45 cycles. This trend suggests that FA/GGBFS mortars retain their compressive strength and become more resilient over time.

The FA mortars initially exhibited a compressive strength of 74.1 MPa at zero cycles. Following 15 cycles, the compressive strength decreased to 58.7 MPa and recovered at 30 cycles, reaching 71.1 MPa. By 45 cycles, the compressive strength slightly decreased to 70.7 MPa, remaining almost equal to the 15-cycle level. This recovery suggests that the compressive strength of FA mortars is not significantly affected by wet–dry cycles. The variation in the measured strength values is likely due to the natural variability of the tested cementitious material.

Similarly, the GGBFS mortars initially exhibited a compressive strength of 75.5 MPa at zero cycles. After 15 cycles, the strength decreased to 62.6 MPa, increased to 81.4 MPa at 30 cycles, and reached a high of 81.3 MPa at 45 cycles. These results highlight the resilience and durability of GGBFS mortars under wet–dry cycles, with limited or no strength degradation.

The control OPC mixes (CEM I 42.5R and CEM I 52.5R) demonstrated different trends, with a significant mechanical degradation with exposure to wet–dry cycles: CEM I 42.5R initially had a compressive strength of 51.8 MPa at 0 cycles, which remained similar after 15 cycles. However, there was a reduction to 31.7 MPa at 30 cycles and further down to 17.1 MPa after 45 cycles. Similarly, CEM I 52.5R initially exhibited a compressive strength of 62.4 MPa at 0 cycles and 70.7 MPa after 15 cycles. Subsequently, the strength decreased to 45.0 MPa at 30 cycles and 28.9 MPa after 45 cycles.

**Figure 8:**
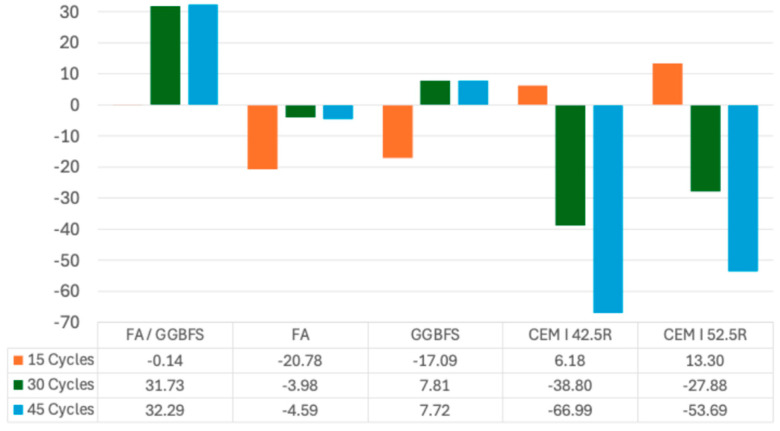
Compressive strength changes (%) under wet–dry cycling.

Figure 8 illustrates the different percentage changes for each mortar type across the three cycle numbers relative to their initial (untreated) compressive strength. The FA/GGBFS mortar exhibited a strength enhancement of 32.95% over 45 cycles. Conversely, the most significant strength degradation occurred in the CEM I 42.5R mortar, showing a 66.6% reduction in its strength after 45 cycles.

The change in the compressive strength results of these mortars subjected to wet–dry cycling in a saline environment can be attributed to the following factors:

- Leaching and degradation: Traditional cement mortars (CEM I 42.5R and CEM I 52.5R) experienced significant declines in their compressive strength over time, such as a decrease from 51.8 MPa at 0 cycles to 17.1 MPa after 45 cycles for CEM I 42.5R. This decline can be attributed to chemical deterioration and leaching of calcium salts.
- Capillary action and moisture uptake: Exposure to wet–dry cycles can cause moisture uptake and loss, potentially leading to microcracks and internal stresses within the mortars. This is more detrimental to traditional cement mortars, which show decreased compressive strength over time.
- Material composition and bonding: The composition and bonding of mortar materials influence their response to cycles. Alkali-activated mortars, particularly those composed of FA/GGBFS and GGBFS, exhibit resilience and improved compressive strength due to their dense structure and strong bonding.

The initial decrease in strength during the first 15 wet–dry cycles in salty water for NaOH-activated fly ash and blast-furnace slag binders is likely due to microcracking from crystallization pressure, differential shrinkage and expansion, the disruption of early hydration and gelation processes, surface damage, and the instability of early reaction products. As the wet–dry cycles progress, the healing of microcracks, pore refinement, stabilization of reaction products, and increased polymerization lead to a recovery of and eventual increase in strength.

The test results show that the alkali-activated mortars demonstrated resilience and improved compressive strength likely due to their chemical and microstructural properties. Traditional OPC mortars, however, experienced declines in compressive strength during treatment due to chemical deterioration and moisture-related damage.

## 4. Conclusions

The objective of this study was to assess the durability properties of heat-cured geopolymer mortars made with FA and GGBFS, activated with sodium hydroxide, after exposure to wet–dry cycling in an aggressive salty solution. The following conclusions can be drawn from the experimental data and comparisons with control specimens:

Water absorption: Geopolymer mortars demonstrated a consistent decrease in water absorption with increasing wet–dry cycles. The FA/GGBFS mortars exhibited a decrease from 6.8 to 4.7% over 45 cycles, the FA mortars from 5.7 to 3.8%, and GGBFS from 4.9 to 2.6%.

Porosity: Decreasing porosity was observed in all geopolymer mortars, signifying enhanced material performance. FA/GGBFS reduced from 14.1 to 9.9%, FA from 11.9 to 8.2%, and GGBFS from 10.5 to 5.5%.

Flexural strength: Although the flexural strength varied among the different types of tested mortars, the flexural strength of geopolymer mixes did not change or showed a slight improvement over the cycles. For example, FA/GGBFS showed a marginal decrease from 7.4 to 7.2 MPa, while GGBFS exhibited an increase from 6.4 to 5.9 MPa.

Compressive strength: Geopolymer mortars demonstrated substantial increases in compressive strength over cycles. The FA/GGBFS mortars increased from 66.5 to 87.9 MPa, FA from 74.1 to 70.7 MPa, and GGBFS from 75.5 to 81.3 MPa.

Control specimens: Control OPC mortar specimens (CEM I 42.5R and CEM I 52.5R) showed significant deterioration under the saline treatment. The compressive strength in CEM I 42.5R decreased from 51.8 to 17.1 MPa, and in CEM I 52.5R from 62.4 to 28.9 MPa.

These experimental results provide additional information and data on the durability of geopolymer mortars in comparison to OPC mortars when subjected to aggressive saline exposure, demonstrating that geopolymer mortars can be regarded as promising alternatives to traditional cement mortars for coastal and marine structures. Future research should explore the long-term performances of geopolymer mortars under various harsh conditions to validate these initial results further.

## Figures and Tables

**Table 1:** Chemical compositions of GGBFS, FA, CEM I 42.5R, and CEM I 52.5R, expressed in %.

Oxide	SiO_2_	Al_2_O_3_	Fe_2_O_3_	CaO	MgO	SO_3_	K_2_O	Na_2_O	LOI
GGBFS	36.7	5.20	0.98	32.61	10.12	0.99	0.76	0.42	2.88
FA	61.81	19.54	7.01	1.77	2.56	0.31	0.99	2.43	2.20
CEM I 42.5R	18.87	5.62	2.54	62.78	2.63	2.82	0.9	0.4	-
CEM I 52.5R	21.6	4.05	0.26	65.7	1.30	3.30	-	-	-

LOI = loss on ignition.

**Table 2:** The mix design and curing conditions of the mortars.

Series	FA (g)	GGBFS (g)	OPC (g)	Water (g)	NaOH (g)	Sand (g)	Curing Temperature (°C)	Curing Period (Days)
FA/GGBFS	255	195	-	180	121	1350	100	3
FA	450	-	-	180	142	1350	100	3
GGBFS	-	450	-	180	150	1350	100	3
CEM I 42.5R	-	-	450	225	-	1350	21 ± 1	28
CEM I 52.5R	-	-	450	225	-	1350	21 ± 1	28

**Table 3:** Test results.

	Number of Wet–Dry Cycles	Water Absorption (%)	COV	Porosity (%)	COV	Flexural Strength (MPa)	COV	Compressive Strength (MPa)	COV
	0	6.8	0.019	14.1	0.022	7.4	0.05	66.5	0.02
FA/GGBFS	15	7.6	0.026	15.6	0.019	8.3	0.09	66.4	0.05
	30	5.8	0.047	12.3	0.041	5.5	0.01	87.6	0.02
	45	4.7	0.048	9.9	0.041	7.2	0.19	87.9	0.06
	0	5.7	0.013	11.9	0.009	11.0	0.15	74.1	0.04
FA	15	6.6	0.015	13.6	0.013	6.0	0.14	58.7	0.03
	30	4.0	0.008	8.4	0.004	4.0	0.04	71.1	0.09
	45	3.8	0.016	8.2	0.020	3.8	0.23	70.7	0.36
	0	4.9	0.028	10.5	0.017	6.4	0.05	75.5	0.07
GGBFS	15	6.2	0.014	13.1	0.038	4.1	0.17	62.6	0.33
	30	3.9	0.008	8.4	0.011	5.5	0.11	81.4	0.04
	45	2.6	0.047	5.5	0.049	5.9	0.17	81.3	0.05
	0	7.9	0.018	16.2	0.005	8.6	0.08	51.8	0.05
CEM I	15	3.6	0.008	8.8	0.005	6.2	0.01	55.0	0.02
42.5R	30	3.9	0.039	8.4	0.025	7.4	0.04	31.7	0.04
	45	3.1	0.029	6.7	0.027	7.4	0.01	17.1	0.16
	0	7.6	0.023	15.8	0.013	10.1	0.13	62.4	0.02
CEM I	15	4.1	0.170	8.9	0.168	11.5	0.06	70.7	0.04
52.5R	30	3.4	0.029	7.4	0.007	7.3	0.03	45.0	0.04
	45	2.9	0.003	6.4	0.002	7.0	0.08	28.9	0.08

COV = coefficient of variation.

## Data Availability

The original contributions presented in the study are included in the article, further inquiries can be directed to the corresponding author.
